# The Role of Vitamin C in Reducing Pain Associated With Diabetic Neuropathy

**DOI:** 10.7759/cureus.15895

**Published:** 2021-06-24

**Authors:** Amerta Bai, FNU Abdullah, Jatender Kumar, Amar Lal, Mohammed Abbas, Ram Sandesh, Sidra Naz, Simra Shahid, Faryal Anees, Sidra Memon

**Affiliations:** 1 Neurology, Jinnah Sindh Medical University, Karachi, PAK; 2 Internal Medicine, Jinnah Sindh Medical University, Karachi, PAK; 3 Internal Medicine, University of Arizona, Tucson, USA; 4 Neurology, University of Louisville, Louisville, USA; 5 Internal Medicine, Ghulam Muhammad Medical College, Sukkur, PAK; 6 Internal Medicine, University of Health Sciences, Lahore, PAK

**Keywords:** vitamin c, diabetes, neuropathic pain, vas, quality of life

## Abstract

Introduction: Neuropathic pain is a painful condition that arises after a lesion or an insult to the somatosensory nervous system, either in a central or peripheral location. The most common cause of neuropathic pain is diabetes. Controlled trials have been conducted on recent advancements in medicine to investigate the effect of vitamin C in the treatment of neuropathic pain. In this study, we aim to investigate the role of vitamin C in reducing pain associated with diabetic neuropathy.

Methods: This open-label, parallel-arm, interventional study was conducted in a public tertiary care hospital in Pakistan from April 2019 to March 2021. A total of 300 type II diabetic patients with newly diagnosed painful peripheral diabetic neuropathy, of either gender, were enrolled in the study. The intervention group received 60 mg duloxetine along with 200 mg oral vitamin C. The control group received 60 mg duloxetine without any additional intervention. Patients were asked to return for follow-up after 12 weeks.

Results: The mean visual analog score (VAS) was significantly lower in both, the intervention (5.54 ± 0.81 vs. 6.72 ± 0.90; p-value: <0.0001) and the control group (5.91 ± 0.80 vs. 6.79 ± 0.94; p-value: <0.0001), at week 12 compared to day 0. However, in comparison, VAS score in intervention at week 12 was significantly lower as compared to the control group (5.54 ± 0.81 vs. 5.91 ± 0.80; p-value: 0.0002).

Conclusion: The use of vitamin C could be cost-effective and would be a safe and useful adjunctive therapy for pain associated with diabetic peripheral neuropathy.

## Introduction

Neuropathic pain is a painful condition that arises after a lesion or an insult to the somatosensory nervous system, either in the central or peripheral location [1]. It affects 7%-10% of the general population, producing a spontaneous ongoing shooting or an evoked pain after a noxious or non-noxious stimulus. It is a broad term that includes various types, such as diabetic neuropathy, post-herpetic neuralgia, trigeminal neuralgia, cancer-related neuropathy, and post-stroke pain [2]. Neuropathic pain may be characterized by hyperesthesia, paresthesia, spontaneous ongoing pain, dysesthesia, hyperalgesia, electric shock-like sensations, allodynia, or mechanical and thermal hypersensitivity. Patients usually have abnormal sensations or hypersensitivity in the affected area [2,3]. Long-term complications of neuropathic pain depend upon the underlying pathology, but in general, if the symptoms persist for long they tend to become chronic and respond less to analgesics. Psychosocial symptoms like depression, anxiety, and sleep disturbances are frequently observed with chronic neuropathic pain, followed by physical disability and poor quality of life (QOL) [1].

Pharmacological therapy is worldwide used as the first line of management of neuropathic pain, followed by interventional strategies, such as nerve blocks and neuromodulators. Antidepressants and antiepileptics have been the most studied drugs in neuropathic pain, including those with confirmed efficacy, like amitriptyline and serotonin-noradrenaline reuptake inhibitors [4]. Various such traditional treatments have been used to improve the QOL of the patients having neuropathic pain, but more than two-thirds of them have obtained insufficient pain relief [5].

Controlled trials have been conducted on recent advancements in medicine to investigate the effect of vitamin C in the treatment of neuropathic pain, especially post-herpetic neuralgia and cancer-related neuropathy [6,7]. The analgesic mechanism of vitamin C owes to its antioxidant and anti-inflammatory properties [8]. It is found to increase the synthesis of catecholamine, dopamine and act as a co-factor for the synthesis of nor-epinephrine, all of which have a significant role in pain relief [7,9].

However, limited data are available on the effectiveness of vitamin C in the long-term control of different types of neuropathic pain, and whether it could reduce the requirement of interventional pain strategies. In this study, we aim to investigate the role of vitamin C in reducing diabetic neuropathic pain.

## Materials and methods

This open-label, parallel-arm, interventional study was conducted in a public tertiary care hospital in Pakistan from April 2019 to March 2021. We enrolled 300 type II diabetic patients with newly confirmed painful peripheral diabetic neuropathy, of either gender, via consecutive convenient non-probability sampling. The study was conducted after approval from the institutional review board. Informed consent was attained from all participants. Biothesiometry was done for all the patients to diagnose and confirm peripheral diabetic neuropathy after clinical and physical examination. Biothesiometry score of 16 volts or above was diagnostic for painful diabetic peripheral neuropathy (PDPN).

After enrollment, participants were randomized into two groups by a 1:1 ratio using an online software research randomizer (https://www.randomizer.org/). The intervention group received 60 mg duloxetine along with 200 mg oral vitamin C. The control group received 60 mg duloxetine without any additional intervention. Patients with diabetic foot, retinopathy, and nephropathy were excluded from the study.

After randomization, patients’ history was taken and their pain was recorded on a visual analog scale (VAS). VAS has a range of 0 to 10, with a higher number indicating greater severity of pain. Patients were asked to return for follow-up after 12 weeks. During follow-up, the pain was recorded again on the VAS scale. The occurrence of adverse events was recorded. Upon follow-up, one patient had stopped taking the medication in the intervention group due to adverse events, while 11 and 13 patients were lost to follow-up in the experiment and placebo group, respectively.

Data were processed through and analyzed using the Statistical Packages for Social Sciences version. 23.0 (SPSS, IBM Corporation, Armonk, NY, USA). Mean and standard deviation (SD) was calculated for continuous variables; frequencies and percentages were calculated for categorical variables. The mean pain score was compared using the t-test. Adverse events in both groups were compared using chi-square. A p-value of less than 0.05 meant that there is a difference between the two groups and the null hypothesis was void.

## Results

In the control group, participants were significantly younger, compared to the interventional group (56 ± 7 years vs 58 ± 7; p-value: 0.01). However, there was no difference in gender distribution and duration of diabetes (Table 1).

**Table 1:** Comparison of demographics of participants NS: nonsignificant, SD: standard deviation

Characteristics	Interventional group (n=138)	Control group (n=137)	P-value
Age in years (Mean ± SD)	58 ± 7	57 ± 8	NS
Gender			
Male	81 (58.6%)	77 (56.2%)	NS
Female	57 (41.4%)	60 (43.8%)	
Duration of diabetes in years (Mean ± SD)	8.01 ± 1.7	7.56 ± 1.5	NS

The mean visual analog score (VAS) was significantly lower in both, the intervention (5.54 ± 0.81 vs. 6.72 ± 0.90; p-value: <0.0001) and the control group (5.91 ± 0.80 vs. 6.79 ± 0.94; p-value: <0.0001), at week 12 compared to day 0. However, in comparison, VAS score in intervention at week 12 was significantly lower as compared to the control group (5.54 ± 0.81 vs. 5.91 ± 0.80; p-value: 0.0002) (Table 2).

**Table 2:** VAS score on day 0 and week 12 *Mean values of day 0 and week 12 within the group were compared **Mean values of week 12 for both groups were compared SD: standard deviation; VAS: visual analog scale

Group	VAS Score (Mean ± SD)		P-value	
	Day 0	Week 12	Intragroup*	Intergroup**
Intervention	6.72 ± 0.90	5.54 ± 0.81	< 0.0001	0.0002
Control	6.79 ± 0.94	5.91 ± 0.80	< 0.0001	

The most common adverse event in both groups was constipation, followed by orthostatic hypotension. There was no significant difference in adverse events between both groups (Table 3).

**Table 3:** Comparison of adverse events between the interventional and control group NA: not available, NS: nonsignificant

Adverse events	Interventional group (n=138)	Control group (n=137)	P-value
Discontinuation due to adverse event	01 (0.72%)	00 (NA)	NS
Constipation	10 (7.25%)	09 (6.57%)	NS
Orthostatic hypotension	05 (3.62%)	06 (4.38%)	NS
Lethargy	04 (2.90%)	04 (2.92%)	NS
Decreased appetite	02 (1.45%)	04 (2.92%)	NS
Blurred vision	01 (0.72%)	01 (0.73%)	NS

## Discussion

The results of our study compared the VAS score between the control and intervention groups. It demonstrated that the VAS score was significantly lower in both groups at week 12 as compared to day 0. However, the reduction was more in the vitamin C group at week 12 as compared to the control group. Moreover, constipation was the most common adverse event in both groups, followed by orthostatic hypotension. Consistent with other studies, the results of our study support the evidence that vitamin C plays a considerable role in reducing pain. In randomized control trials, vitamin c supplementation showed a decline in the incidence of complex regional pain syndrome [10,11]. Similarly, in a study conducted by Schencking et al., patients with herpes zoster reported a great decline in pain after receiving vitamin C [12]. In addition to this, two prospective studies of patients with advanced cancer also showed a decline in pain within one to four weeks after taking vitamin C doses [12,13].

Vitamin C tends to protect cells and tissues from oxidative damage. It is considered to be a potent antioxidant and can eradicate a wide range of reactive oxygen species. It is assumed to act in oxidative stress conditions because of its well-known oxidative stress properties [14]. It also has anti-inflammatory properties by decreasing the markers of inflammation such as C-reactive protein and pro-inflammatory cytokines, e.g. tumor necrosis factor, interferon, and interleukin [15]. Due to its enzymatic properties, it also acts as a cofactor in our body for the synthesis of catecholamine neurotransmitters; hence, highlighting its role in neuromodulation [16]. Vitamin C also increases the amidated neuropeptide hormones in humans during severe infections. Therefore, due to the sub-optimal biosynthesis of analgesic neurotransmitters and neuropeptide hormones, decreasing vitamin C contributes to pain symptoms during acute or chronic disease [17]. Oral vitamin C is transported through intestinal epithelium and due to the saturation of transporters, it becomes less efficient [18]. In contrast, intravenously administered vitamin C, which bypasses the intestinal epithelium, can provide plasma concentrations that are 250-fold higher [19]. Therefore, to make it more efficient and to maximize the plasma concentration, an intravenous dose should be ideally administered in several smaller doses over the day [20]. The use of opioid analgesics is an essential component in the management of pain worldwide and is associated with both therapeutic and adverse effects [21].

The study has its limitation. First, since the study was conducted in a single institute, the sample size was less diverse. Second, participants’ compliance with medication was not monitored, which might have impacted the result.

## Conclusions

Vitamin C plays a synergistic role in reducing pain in patients with diabetic neuropathy. It improves QOL due to its analgesic properties. Moreover, it is cost-effective and appears to be a safe and adjuvant therapy for specific pain relief. Patients should be encouraged to take vitamin C along with pharmacological management to get maximum relief from pain.
